# Challenges to Rehabilitation Services in Sub-Saharan Africa From a User, Health System, and Service Provider Perspective: Scoping Review

**DOI:** 10.2196/58841

**Published:** 2025-02-28

**Authors:** Callixte Cyuzuzo, Marie Josee Dukuzimana, Clement Muhire, Mathew Sheldon Ames, Emmanuel Ngwakongnwi

**Affiliations:** 1 Center for Population Health, Institute of Global Health Equity Research, University of Global Health Equity, Kigali, Rwanda; 2 Institute of Global Health Equity Research, University of Global Health Equity, KG 7 Ave, 5th Floor, PO Box 6955, Kigali, Rwanda, 250 785690861; 3 Campus Operations and Community Engagement, University of Global Health Equity, Burera, Rwanda; 4 Educational Development and Quality Center, University of Global Health Equity, Kigali, Rwanda

**Keywords:** challenges, users, health system, service providers, Sub-Saharan Africa, scoping review, rehabilitation service

## Abstract

**Background:**

Rehabilitation aims to restore and optimize the functioning of impaired systems for people with disabilities. It is an integral part of universal health coverage, and access to it is a human right.

**Objective:**

We aimed to identify the key challenges to rehabilitation services in Sub-Saharan Africa from a user, health system, and service provider perspective.

**Methods:**

This scoping review was conducted in accordance with the 5-stage framework proposed by Arksey and O’Malley. A comprehensive electronic search was run to identify published articles on rehabilitation services in Sub-Saharan Africa. Of the 131 articles retrieved, 83 articles were assessed for eligibility and 15 papers that met the inclusion criteria were considered.

**Results:**

The results show that people with disabilities in Sub-Saharan Africa face multifactorial challenges to access rehabilitation services. Poor access to rehabilitation services is associated with less attention given to rehabilitation by governments, which leads to less funding, negative cultural and social beliefs, fewer rehabilitation centers, poorly equipped rehabilitation units, failure of health systems, lack of training to rehabilitation practitioners, and logistical and financial constraints. This review also reveals that digital rehabilitation reduces costs and improves access to services in hard-to-reach geographical areas. However, digital rehabilitation faces challenges as well, including connectivity issues, inaccessibility to technology, a lack of technical knowledge, a lack of privacy, and ethical concerns.

**Conclusions:**

People with disabilities face multifactorial challenges to access rehabilitation services in Sub-Saharan Africa. It is therefore critical to address these challenges to optimize patients’ health outcomes and offer better rehabilitation services.

## Introduction

Rehabilitation refers to a set of therapeutic approaches that are structured to address physical, developmental, emotional, and mental challenges and improve patients’ health outcomes and quality of life [1]. Rehabilitation is an integral part of universal health coverage and contributes greatly to the achievement of the United Nations’ Sustainable Development Goal 3, which aims to ensure healthy lives and promote well-being for people of all ages [2]. Access to rehabilitation for people with disabilities is a human right, as stated in Article 26 of the United Nations Convention on the Rights of Persons with Disabilities [3]. Rehabilitation services can be either delivered through traditional face-to-face methods or, using remote approaches, facilitated by existing technologies [4,5]. Digital rehabilitation has reduced costs and improved access to rehabilitation services in hard-to-reach geographical areas [5].

Demographic changes that lead to chronic health conditions and accidents have gradually contributed to the increased need for rehabilitation services [6]. It is estimated that 2.4 billion people have a health condition that may benefit from rehabilitation services; however, these services are still inaccessible due to a shortage of rehabilitation practitioners [1]. A World Health Organization (WHO) report disclosed that the ratio of skilled rehabilitation practitioners to patients is 10 to 1,000,000 in low- and middle-income countries. This leads to a persistent scarcity of health-related human resources and reduced access to rehabilitation services in low-resource regions [7].

Sub-Saharan Africa (SSA) has a shortage of health-related human resources, with the region accounting for only 3.5% of the world’s health workers, despite it having a high disease burden that includes several kinds of disabilities [1]. In this low-resource region, there is a significant difference in the availability of and access to rehabilitation services, and this impedes individuals with disabilities from achieving their desired health outcomes [8,9]. The WHO reported that in SSA, 50% of people do not get the rehabilitation services they need, and a high proportion of people with disabilities with unmet needs are in low- and middle-income countries [10].

Demand for rehabilitation services in SSA is well established given the prevalence of unmet rehabilitation needs, the rising cases of noncommunicable diseases, and the significant incidence of road traffic accidents that result in disabilities. Additionally, lack of access to rehabilitation services for people with chronic conditions can lead to a need for assistance with activities of daily living and long-term hospital stays [11]. It is important to explore the rehabilitation challenges faced by users, health systems, and service providers. This scoping review aims to enhance our understanding of the complex demands imposed by these factors in SSA and examine the challenges associated with rehabilitation services from those 3 domains.

## Methods

### Study Design

This review follows the framework of conducting scoping reviews as proposed by Arksey and O’Malley [12]. This framework consists of 5 distinct stages: identifying the research question; identifying relevant studies; selecting studies; charting the data; and collating, summarizing, and reporting the results.

#### Identifying the Research Question

The research topic and its associated objectives helped to determine the scope of the review, appropriate literature, search strategy, and inclusion and exclusion criteria. To attain the main aim of this review, the scope of rehabilitation services in SSA was explored and key challenges that users, service providers, and health systems face on the availability of and access to rehabilitation services were identified.

#### Identifying Relevant Studies

##### Search Strategy

A comprehensive electronic search was conducted to retrieve published data on the availability of and access to rehabilitation services in SSA. Research studies published in reputable journals and databases such as PubMed, National Center for Biotechnology Information, Scopus, PLOS, BioMed Central, Taylor and Francis, Science Direct, Frontiers, Springer Nature, and Web of Science were chosen, as they yielded the most topic-relevant articles. A search strategy using the keywords “rehabilitation services,” “availability,” “access,” and “Sub-Saharan Africa” was used. To increase the number of accessed literatures, “Sub-Saharan Africa” was replaced by the name of the country, and “rehabilitation services” was replaced by specific types of rehabilitation services such as physiotherapy, occupational therapy, speech and language therapy, and prosthetics services. To maximize the literature coverage and to provide additional evidence, reference lists of primary studies and review articles were screened to identify additional relevant literature.

##### Inclusion and Exclusion Criteria

Inclusion criteria were developed to ensure that all relevant publications focusing on availability of and access to rehabilitation services in SSA were included. Works written in English and published in the above-mentioned reputable journals and databases between 2018 and 2023 were considered. Articles not written in English, published before 2018, and that did not report on rehabilitation services in SSA countries were excluded.

### Study Selection

One team member led the search and screened the titles and abstracts of all retrieved papers based on the inclusion and exclusion criteria. Articles retained for review were confirmed by the research team after discussion. Duplicates were removed and the articles that satisfied the inclusion criteria were selected for full-text review.

### Charting the Data

With the help of Microsoft Excel, data on availability of and access to rehabilitation services were extracted from the full texts. Challenges faced by users, service providers, and health systems were recorded. Information such as the title of the paper, names of authors, year of publication, study setting, study design, objectives of the paper, inclusion criteria, and study findings were extracted and recorded in the Excel table. Reference lists of included papers were also screened to optimize the search. The data extraction document is attached as a supplementary file (Multimedia Appendix 1).

## Results

### Overview

**Figure 1.**
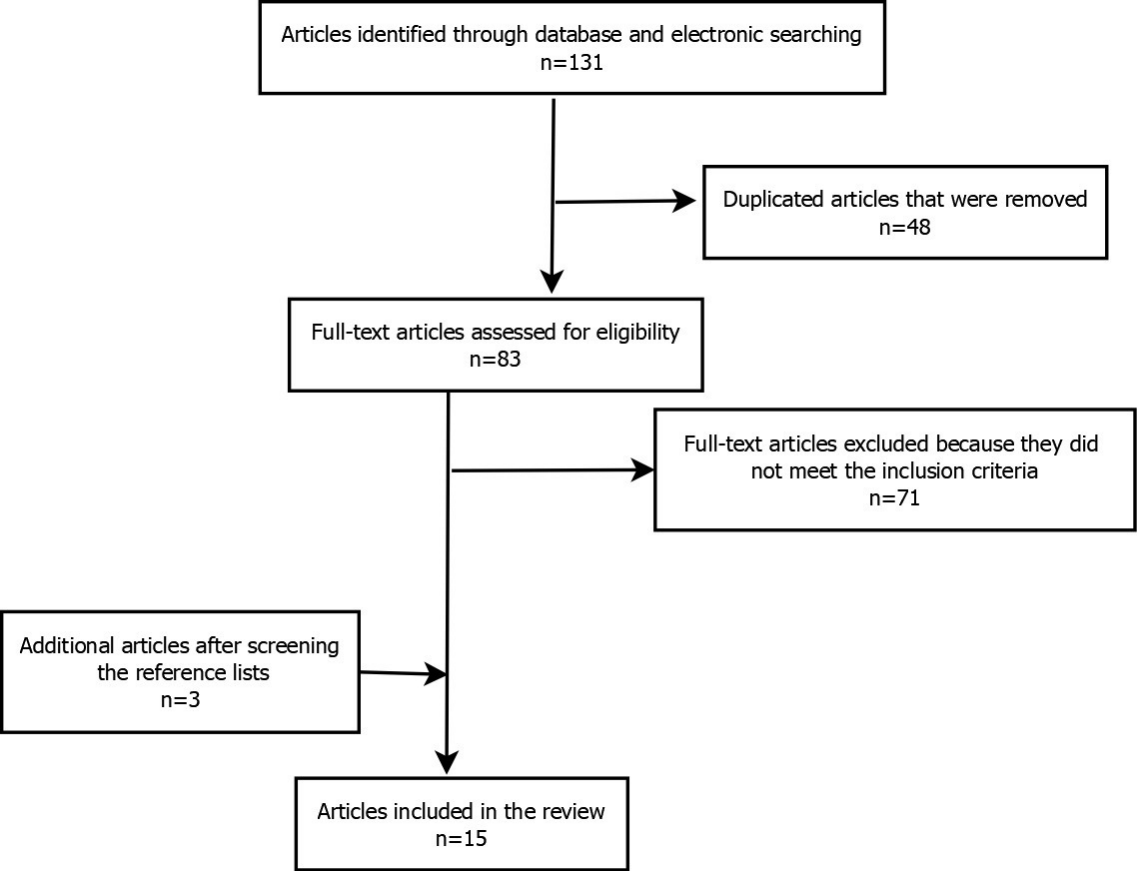
Preferred Reporting Items for Systematic Reviews and Meta-Analyses (PRISMA) flowchart illustrating the article selection process.

The search yielded 131 research and review articles overall. Forty-eight articles were duplicates and 71 articles did not satisfy the inclusion criteria. The full texts of the remaining 12 articles were screened, and 3 more articles were identified using the reference lists, resulting in a total of 15 articles eligible for analysis. Six of the 15 (40%) articles were original studies while 9 (60%) were scoping or systematic reviews covering other aspects of rehabilitation. Figure 1 illustrates how the articles were selected. All 15 considered articles indicated the existence of rehabilitation challenges; of these, 6 papers discussed the challenges faced by users, 5 discussed those faced by health systems, 3 discussed those faced by service providers, and 1 review article reported challenges for all.

### Challenges to Rehabilitation Services in SSA

A synthesis of the literature revealed 2 categories of challenges to rehabilitation services from user, service provider, and health system perspectives (Table 1).

**Table 1.** Challenges to rehabilitation services reported in studies involving users, providers, and health systems.

	Category	
	Accessibility	Availability
Users	Overall challenges: Unsuitable environment and buildings for people with disabilities Inaccessibility to technology Stigma, negative attitudes, and discrimination from therapists and other hospital staff Long queues and waiting time Financial constraints to purchasing medicines Mindset that nothing can be done to help them Lack of psychological support Nonsupportive family members Lack of trust in service providers Pain during the rehabilitation process Religious beliefs and socially accepted norms and traditions that claim that disability results from witchcraft or divine origins Language barriers Digital rehabilitation–specific challenges: Lack of secure platforms and privacy Lack of technical knowledge or digital literacy Connectivity issues Inconsistency in power supply	Do not know where to find the services Long distance between users’ homes and health facilities Lack of reliable transport and logistical affordability Distant health units
Service providers	Overall challenges: Insufficient time for consultation Inadequate follow-up Inappropriate treatment procedures Lack of patience Ethical challenges Digital rehabilitation–specific challenges: Lack of technical knowledge or digital literacy Connectivity issues	Lack of communication skills Lack of professional training and necessary skills Lack of maintenance for the assistive tools Failure to schedule online appointments
Health systems	Overall challenges: Consideration of rehabilitation as a less important health care strategy that is not well integrated into services Fragmented health services Lack of community awareness on rehabilitation services Failure of the health system Digital rehabilitation–specific challenges: Financial constraints	Irregular referral to well-equipped health facilities Lack of medicines and services Shortage of health-related human resources Low attention from governments and underfunding Limited rehabilitation centers and poorly equipped rehabilitation units

## Discussion

### Principal Findings

This review revealed that people with disabilities in SSA have limited access to rehabilitation services. The limited access can be explained partly by user, service provider, and health system factors [8,13]. Studies included in this review [5,14] found that rehabilitation was given less priority by governments and not recognized as a crucial health care service. Consequently, the sector faced underfunding; experienced a deficiency in health-related human resources, particularly in therapists; encountered a scarcity of rehabilitation centers; and witnessed inadequately equipped rehabilitation units within health facilities. This contributed to the compromised access to high-quality rehabilitation services [5,14,15].

Another study reported that limited access to rehabilitation services in SSA is attributed to the financial inability to afford treatment, lack of insurance covering service expenses, long waiting times at the health facilities, and lack of drugs at health facilities [10]. Other studies have also found that some patients lack psychological support, are unaware of where to find services, believe that nothing could be done to help them, and are not involved in making decisions on their health [8,16].

This review identified stigma as an impediment to accessing rehabilitation services. A study conducted in Rwanda [17] found that stigma around disability, coupled with negative attitudes and discrimination from therapists and other hospital staff, negatively impacted the uptake of rehabilitation services. Likewise, the role of family in accessing rehabilitation services is well documented. Patients with family members who do not perceive the need for rehabilitation services and who do not trust service providers may be demotivated to seek rehabilitation services [13,17,18]. Additionally, compromised access to health information, irregularities in referral to health facilities that are well equipped, inadequate policies and standards that govern the services, lack of adequate follow-up, insufficient time for consultations, and pain during the rehabilitation process are all hindrances to accessing rehabilitation services [9,15].

This review found that people with disabilities in SSA lack reliable, affordable, and accessible transportation means to reach health facilities. Furthermore, long distances to services and health units, unsuitable environments, and a lack of wheelchair-accessible buildings prevent users from accessing rehabilitation services. A review conducted in Brazil documented patients’ determinants (residential location, economic resources, and social characteristics) and characteristics of services (cost, location, and status of the available facilities) as factors that limit access to rehabilitation services [8].

The results published by Bright et al [18] revealed that compromised health systems, lack of professional training and necessary skills for therapists, inappropriate treatment procedures, and lack of maintenance for the assistive tools reduce access to high-quality rehabilitation services. Health care providers who have not received enough training may lack the skills required to provide successful rehabilitation services. This lack of competence implies that misdiagnoses or outdated and inappropriate techniques could be used, causing harm to patients.

The study conducted by Jones et al [5] on telerehabilitation reported how digital rehabilitation services have addressed some logistical challenges; however, they come with additional challenges, including the absence of secure platforms and privacy, inaccessibility to technology, and lack of technical knowledge for both users and therapists [8]. Other challenges identified include a failure to schedule online appointments, connectivity issues, ethical challenges, and failure of the health systems. Patients and health care providers may struggle to use digital tools for rehabilitation, limiting the effectiveness of remote health services. Additionally, the inability to schedule and failure to maintain digital appointments may result in missed sessions, decreased patient participation, and care delays. Health systems face the challenge of poor policies that lead to a lack of financial capacity to afford digital technologies in health service delivery [19]. Digital technologies such as online medical records, wearable devices, and telemedicine can improve patient care and accessibility to health services, particularly in hard-to-reach areas. When financial limitations prevent the use of these technologies, patients may not receive the necessary high-quality care on time.

This review discerned that religious beliefs, cultural norms, and traditions that attribute disabilities to witchcraft or divinity serve as deterrents for individuals with disabilities seeking rehabilitation services at health facilities. A study conducted in Rwanda found that people with mental disorders believe that traditional and faith healers are more effective at treating mental problems than hospital specialists [20]. Such cultural beliefs dissuade people with disabilities from rehabilitation services, casting doubt on their quality and efficacy. Uncertainties regarding the precise origins of their conditions, which are often intertwined with mystical beliefs, add to this dissuasion [21].

Considering the rehabilitation challenges identified in this review, there is a need to bridge the gaps of infrastructure and social and cultural awareness regarding disability and rehabilitation services. This should be done by decentralizing services, providing continuous professional training to therapists, and promoting regular community awareness about rehabilitation services to inform service seekers and break the stigma around it. While this review has enumerated the challenges to rehabilitation services in SSA, it is worth noting that a majority of articles retained for consideration were scoping and systematic reviews that examined other aspects of rehabilitation. User and health system challenges were mostly highlighted in comparison to service provider challenges, which were limited in content.

### Conclusion

This review reveals that rehabilitation services in SSA face multifactorial challenges that negatively impact timely access and quality of rehabilitation services for people with disabilities. As a consequence, those who require rehabilitation services experience longer periods of decreased mobility and functioning, inferior quality of life, and lower socioeconomic well-being. Future studies should examine the application of digital technologies to improve rehabilitation services’ accessibility, especially in remote settings.

## Supplementary material

10.2196/58841 Multimedia Appendix 1 Data extraction table.

10.2196/58841 Checklist 1 Preferred Reporting Items for Systematic Reviews and Meta-Analyses extension for Scoping Reviews (PRISMA-ScR) checklist.

## Abbreviations

SSA: Sub-Saharan Africa
WHO: World Health Organization
