# Supplementary material for: Challenges to Rehabilitation Services in Sub-Saharan Africa From a User, Health System, and Service Provider Perspective: Scoping Review
Source: JMIR Hum Factors. 2025 Feb 28;12:e58841. doi: 10.2196/58841 (PMC11887585; doi:10.2196/58841)
Supplement: Multimedia Appendix 1 [file humanfactors-v12-e58841-s001.docx]

|  |  | **Data Extraction Table** | |  |  |  |
| --- | --- | --- | --- | --- | --- | --- |
|  | **Title** | **authors and year of publication** | **Country** | **Sample** | **Study variables** | **Outcome** |
| 1 | Mapping evidence of community health workers delivering physical rehabilitation services in sub-Saharan Africa: a scoping review protocol | Mapulanga & Dlungwane,2022 | South Africa | N/A | Scope and evidence of physical rehabilitation services, mode of delivery, barriers and factors that facilitate the delivery of physical rehabilitation services | Disclosed gaps in rehabilitation services implementation, physical rehabilitation services training for CHWs, increased access and coverage of rehabilitation services, increased body of knowledge on the provision of rehabilitation services. |
| 2 | Scoping review of rehabilitation care models for post COVID-19 condition | Décary et al.,2022 | Canada | N/A | Evidence about health system, provider’s and patients’ characteristics | Multiprofessional and multilevel rehabilitation care model, leveraging available strengths to provide appropriate rehabilitation services, policy making and financing rehabilitation sectors. |
| 3 | Scoping review of remote rehabilitation (telerehabilitation) services to support people with vision impairment | Jones et al.,2022 | United Kingdom | Patients aged 18 years and above with visual impairment caused by both medical and non-medical trauma. | Type of telerehabilitation services available to people with vision impairment and evidence on health-related outcomes, wellbeing and cost effectiveness | Patient satisfaction,  cost-effectiveness, objective visual function, quality-of-life, activities of daily living and well-being |
| 4 | Global Need for Physical Rehabilitation: Systematic Analysis from the Global Burden of Disease Study 2017 | Jesus et al.,2019 | Portugal | N/A | Estimate year lived disability benefiting from physical rehabilitation, changes in estimates over time, difference in trends across countries of varying income levels? | Year lived disability benefits from Physical rehabilitation services, disclosed physical rehabilitation needs and how these needs have been growing over time across locations especially in countries of lower income level with deprived rehabilitation infrastructure. |
| 5 | Access to health and rehabilitation services for persons with disabilities in Sierra Leone - focus group discussions with stakeholders | Magnusson et al.,2022 | Sierra Leone | Seven focus group discussions were conducted with 37 stakeholders working within disability. | Stakeholders’ perceptions of access to health and rehabilitation services for persons with disabilities | Continuous stigmatization of persons with disabilities, long distances and transportation issues to access health and rehabilitation facilities; financial constraints; infrastructural barriers to healthcare and rehabilitation services, healthcare personnel’s negative attitudes and inadequate knowledge towards disabled people, lack of materials to provide quality services; and the need for continuous education of new and current rehabilitation personnel. |
| 6 | Barriers to the access of people with disabilities to health services: a scoping review | Clemente et al., 2022 | Brazil | N/A | Barriers to access health services for the people with disabilities | Communication failure between professionals and patient, financial limitations, attitudinal/behavioral issues, scarce service provision, organizational and transport barriers, lack of training to professionals; failure of the health system; physical barriers; lack of resources/technology; and language barriers. |
| 7 | Barriers and facilitators to utilisation of rehabilitation services amongst persons with lower-limb amputations in a rural community in South Africa | Naidoo & Ennion, 2019 | South Africa | 11 sampled participants from three sub-district hospitals in the rural  iLembe district, Kwa-Zulu Natal, South Africa. | Barriers and facilitators to accessing rehabilitation services experienced by persons with lower limb  amputations in a rural setting. | Identified barriers were environmental factors, financial constraints and impairments. Facilitators identified were  environmental facilitators and personal factors which aided participant’s utilization of rehabilitation. |
| 8 | Building PRM in sub-Saharan Africa | Tannor et al.,2022 | Ghana | N/A | Successes and challenges to improving access to physical rehabilitation medicine care in Sub-Saharan Africa | Identified success is the establishment of International Rehabilitation Forum and the identified challenges are inadequate physical rehabilitation medicine trainers, unavailability of logistics and services for hands on experience, and funding. |
| 9 | A systematic review of access to rehabilitation for people with disabilities in low-and middle-income countries | Bright et al., 2018 | United Kingdom | N/A | Access to rehabilitation for people with disabilities in LMIC (low and middle income countries), with a focus on health-related rehabilitation | Access to rehabilitation services is low among people with disabilities. |
| 10 | Systematic Review on End-Users’ Perception of Facilitators and Barriers in Accessing Tele-Rehabilitation Services | Padmavathi et al., 2023 | India | N/A | End-user’s perception of facilitators and barriers in accessing psychiatric tele-rehabilitation services. | The facilitators include an internet-enabled device, cost/financial benefits, e-healthcare knowledge, technology as a valuable and accessible tool, motivational factors, satisfaction and willingness affordability to the internet enabled device, network connectivity, lack of technical skills, and digital literacy. |
| 11 | Rehabilitation workforce descriptors: a scoping review | Conradie at al.,2022 | South Africa | N/A | Descriptors and metrics that describe the rehabilitation workforce and compare the workforce across countries. | The assessed descriptors and indicators revealed a large disparity in the rehabilitation workforce capacity between HICs and LMICs (low and middle income countries). LMICs have poor access to rehabilitation services and this lack of workforce capacity exacerbates access to rehabilitation services for the most vulnerable populations. |
| 12 | A review of the framework and strategy for disability and rehabilitation services in South Africa | Hussein El Kout et al., 2022 | South Africa | 12 informants were selected purposively. | The barriers and facilitators that influenced the process  of development, implementation and monitoring of the framework and strategy for  disability and rehabilitation services. | Factors that impeded successful implementation of the framework are the dynamics of actors, insufficient resources, negative attitudes of staff members and insufficient monitoring. Positive attitude, mentorship and support  facilitated the implementation process. |
| 13 | Stroke rehabilitation services in Africa – Challenges and opportunities: A scoping review of the literature | Tawa et al., 2021 | South Africa | N/A | Capacity development to complement stroke rehabilitation in Africa. | Stroke rehabilitation services in Africa are poor due to the lack of required components of an effective healthcare service and this impedes the integration of disabled people in the society and community. |
| 14 | Provision and use of physical rehabilitation services for adults with disabilities in Rwanda: A descriptive study | Kumurenzi et al., 2022 | Rwanda | This study considered 213 adult respondents aged 18 years and above with disabilities across 9 facilities | Facilitators and barriers that adults face while seeking rehabilitation services at health facilities. | The availability of rehabilitation services for Rwandan adults with physical disabilities is limited and the transportation costs remain a significant barrier. These challenges could be addressed by developing triage protocols that could be implemented at the referral stage to prevent a constant flow of patients, long waiting lists and time |
| 15 | Barriers and facilitators to cultural competence in rehabilitation services: A scoping review | (Grandpierre et al., 2018) | USA (United States of America) | N/A | Barriers and facilitators to cultural competence in rehabilitation services | Barriers in rehabilitation services include language barriers, limited resources, and cultural barriers. Identified facilitators are cultural awareness amongst practitioners, cultural awareness in services, and explanations of health care systems. |
